# Bioactive 2-(Methyldithio)Pyridine-3-Carbonitrile from Persian Shallot (*Allium stipitatum* Regel.) Exerts Broad-Spectrum Antimicrobial Activity

**DOI:** 10.3390/molecules24061003

**Published:** 2019-03-13

**Authors:** Arunkumar Karunanidhi, Ehsanollah Ghaznavi-Rad, Jayakayatri Jeevajothi Nathan, Narcisse Joseph, Sridevi Chigurupati, Fazlin Mohd Fauzi, Mallikarjuna Rao Pichika, Rukman Awang Hamat, Leslie Than Thian Lung, Alex van Belkum, Vasanthakumari Neela

**Affiliations:** 1 Department of Medical Microbiology and Parasitology, Faculty of Medicine and Health Sciences, Universiti Putra Malaysia, Serdang 43400, Selangor Darul Ehsan, Malaysia; arunmscimb@gmail.com (A.K.); narcissemsjoseph@gmail.com (N.J.); rukman@upm.edu.my (R.A.H.); leslie@upm.edu.my (L.T.T.L.); 2 Department of Pharmacology and Chemistry, Faculty of Pharmacy, Universiti Teknologi MARA, Bandar Puncak Alam 42300, Selangor Darul Ehsan, Malaysia; fazlin205@gmail.com; 3 Department of Microbiology and Immunology, Faculty of Medicine, Arak University of Medical Sciences, Basij Square, Arak 38481-7-6941, Iran; ghaznaviehs@yahoo.com; 4 School of Medicine, Taylor’s University, Lakeside Campus, No. 1, Jalan Taylor’s, Subang Jaya 47500, Selangor Darul Ehsan, Malaysia; jjayakayatri@yahoo.com; 5 Department of Medicinal Chemistry and Pharmacognosy, College of Pharmacy, Qassim University, Buraidah 52571, Kingdom of Saudi Arabia; sridevi.phd@gmail.com; 6 Department of Pharmaceutical Chemistry, School of Pharmacy, International Medical University, 126 Jalan Bukit Jalil, Kuala Lumpur 57000, Malaysia; mallikarjunarao_pichika@imu.edu.my; 7 R & D Microbiology, BioMerieux, 3 route de Port Michaud, 38390 La Balme-les-Grottes, France; alex.vanbelkum@biomerieux.com

**Keywords:** *Allium stipitatum*, antibacterial, minimum inhibitory concentration, methicillin-resistant *Staphylococcus aureus*

## Abstract

Antibiotic resistance is a problem that continues to challenge the healthcare sector, especially in clinically significant pathogens like methicillin-resistant *Staphylococcus aureus* (MRSA). Herein is described the isolation and structure elucidation of a bioactive compound from *Allium stipitatum* with antimicrobial activity. Crude *Allium stipitatum* dichloromethane extract (ASDE) was subjected to systematic purification by chromatographic procedures to afford various bioactive fractions. A fraction that exhibited anti-MRSA activity (4 µg·mL^−1^) was further characterized to determine the structure. The structure of the compound was elucidated as 2-(methyldithio)pyridine-3-carbonitrile (2-Medpy-3-CN). The 2-Medpy-3-CN compound, which was screened for antimicrobial activity, exhibited minimum inhibitory concentrations (MICs) in the range of 0.5 to >64 µg·mL^−1^ for tested bacterial species and 0.25 to 2 µg·mL^−1^ for *Candida* spp. Further studies are important to confirm the drug target and mechanism of action.

## 1. Introduction

The sustained increase in life-threatening infectious diseases and emerging infectious diseases (EID) due to the multidrug-resistant (MDR) pathogens is an immense and serious global challenge. Infections associated with MDR *Staphylococcus aureus*, including methicillin-resistant *S. aureus* (MRSA) and vancomycin-resistant *S. aureus* (VRSA) [1], and with vancomycin-resistant *Enterococcus faecium* [2], MDR *Acinetobacter baumannii* [3], trimethoprim/sulfamethoxazole (TMP-SMX)-resistant *Stenotrophomonas maltophilia* [4], and azole-resistant *Candida albicans* [5,6] are adequately reported worldwide.

Among the many clinically challenging microbes, *S. aureus* is a well-armed virulent pathogen that is currently the most common cause of hospital- and community-acquired infections worldwide. Its increasing resistance to antibiotics indicates that its prevalence will continue to rise. In addition, the high morbidity and mortality rates [7], and high treatment costs in combating MRSA infections [8,9,10] strongly underscore the pressing need for new classes of antimicrobials to overcome multidrug resistance.

There are a vast number of natural products that are a likely source of novel antibiotic compounds [11]. In particular, plants serve as main source of bioactive substances including antibacterial compounds, and there are noteworthy studies supporting their preliminary in vitro efficacy [12,13,14]. *Allium* has a long history of its use in traditional medicine with high therapeutic properties and its ethno-medicinal use is reported from most regions of the temperate world including Asia. An extensive review by Petrovska and Cekovska [15] clearly showcased the use of garlic (*Allium sativum* L.) for human health from the ancient times until today. Garlic was widely used as an antiseptic to prevent gangrene in the First and Second World Wars [15,16]. Several edible products including different species of *Allium* (garlic, onion, shallots) were shown to harbor remarkable antibacterial activities [17,18,19].

In our earlier study, we identified dichloromethane extract of *Allium stipitatum* or Persian shallot, popularly known as “Mooseer”, a wild edible plant mostly found in the cold mountains of central, south, and western Iran, some provinces of Turkey, and central Asia, to exhibit strong antimicrobial and wound-healing activity [20,21,22,23,24]. Pyridine-*N*-oxide alkaloids and analogs of disulfides from *A. stipitatum* were reported to possess moderate to strong antibacterial activity against MDR *S. aureus*, *Mycobacterium tuberculosis*, *Escherichia coli*, and *Klebsiella* and *Proteus* species [25,26]. The methyl disulfide analogs were also reported to inhibit the mycobacterial drug efflux systems and biofilm formation in *M. tuberculosis* H37Rv strains [25]. These data indicates that *A. stipitatum* retains intrinsic antimicrobial properties that likely contribute to the positive outcomes observed in in vivo and in vitro studies.

The promising in vitro and in vivo antimicrobial activities prompted us to explore the isolation of bioactive compounds from the bulbs of *A. stipitatum*. Therefore, the present study was aimed at purifying the bioactive compound from *A. stipitatum* that exhibits antimicrobial activity.

## 2. Results

### 2.1. Bioassay-Guided Fractionation

A total of 80 fractions were obtained through silica gel column chromatography (CC) and all the fractions were subjected for thin-layer chromatography (TLC) analysis. Fractions that displayed similar TLC patters were pooled together and divided into six major fractions (D_1_–D_6_). Screening for antibacterial activity of fractions D_1_–D_6_ showed fraction D_3_ to have strong anti-MRSA activity with a minimum inhibitory concentration (MIC) of 32 µg·mL^−1^. The MICs of fractions D_2_ and D_4_ were 128 µg·mL^−1^ and 256 µg·mL^−1^, respectively. However, fractions D_1_, D_5_, and D_6_ did not show any activity (Table 1). Fraction D_3_ which was subjected to further purification by preparative TLC (PTLC) showed eight major fractions (D_3/1_–D_3/8_). Screening for antibacterial activity of fractions D_3/1_–D_3/8_ showed fraction D_3/6_ to have strong anti-MRSA activity with an MIC of 16 µg·mL^−1^. The MICs of fractions D_3/5_ and D_3/6_ were 16 µg·mL^−1^, while the MICs of fractions D_3/4_ and D_3/7_ were 32 µg·mL^−1^. Fractions D_3/1_–D_3/3_ and D_3/8_ did not show any activity (Table 2). Fraction D_3/6_ was separated into 120 subfractions by CC, which were pooled into six subfractions (D_3/6a_–D_3/6f_) based on the TLC patterns. Screening for antibacterial activity of subfractions D_3/6a_–D_3/6f_ showed D_3/6e_ to have strong anti-MRSA activity with an MIC of 4 µg·mL^−1^. Subfraction D_3/6d_ inhibited the growth of MRSA at 32 µg·mL^−1^, while subfractions D_3/6a–c_ and D_3/6f_ did not show any activity (Table 3).

### 2.2. Structure Elucidation and Identification

**Figure 1:**
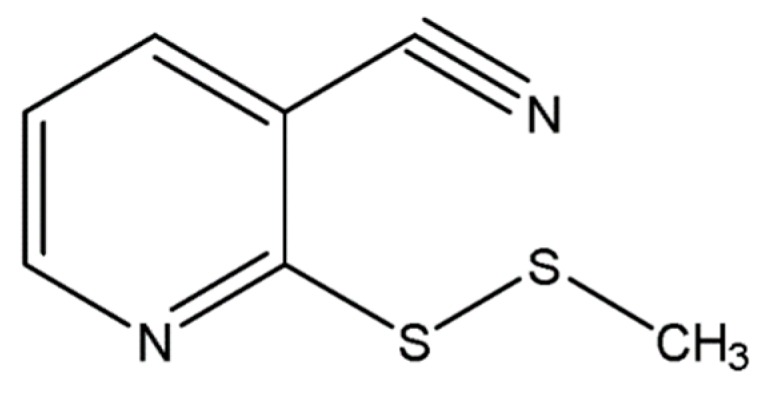
Two-dimensional (2D) structure of 2-(methyldithio)pyridine-3-carbonitrile (2-Medpy-3-CN).

Fraction D_3/6e_ was identified as 2-(methyldithio)pyridine-3-carbonitrile (2-Medpy-3-CN) (Figure 1) based on spectroscopic methods.

*2-Medpy-3-CN:* yellow crystalline; ultraviolet (UV) (MeOH) λ_max_ nm (log *ε*): 221 (1.19), 272 (2.35); yield 85.86%; melting point (m.p.) 70.6–71.4 °C; infrared (IR) (KBr, disc) ν_max_ cm^−1^: 3064, 2911, 2221, 1567, 1544, 1430, 1383, 1258, 1230, 1128, 1066, 957, 731; positive LC–MS/MS *m/z* 183.0047 [M + H]^+^, (calculated for C_7_H_6_N_2_S_2_, 182.27); *m*/*e*: 182 (100.0%), 183 (9.3%), 183.99 (9.5%); ^1^H-NMR (500 MHz, CDCl_3_) and ^13^C-NMR (125 MHz, CDCl_3_) spectral values were recorded in one-dimensional (1D) and two-dimensional (2D) models (Table 4).

### 2.3. Minimum Inhibitory Concentration (MIC), Minimum Bactericidal Concentration (MBC), and Minimum Fungicidal Concentration (MFC) of 2-Medpy-3-CN

Based on the susceptibility testing results obtained, 2-Medpy-3-CN exhibited strong antibacterial activity against six out of nine pathogens tested. The MICs of 2-Medpy-3-CN ranged from 0.5 to >64 µg·mL^−1^ (Table 5). Gram-positive MSSA (methicillin-sensitive *S. aureus*) and MRSA and Gram-negative *Acinetobacter iwoffii* and *Acinetobacter baumannii* were highly susceptible to 2-Medpy-3-CN. The lowest MIC was observed for *A. iwoffii* (0.5 µg·mL^−1^), while *A. baumannii*, MRSA, and MSSA showed MICs of 4 µg·mL^−1^. *Escherichia coli* and *Stenotrophomonas maltophilia* were susceptible at slightly high concentration (32 µg·mL^−1^). However, *Pseudomonas aeruginosa*, *Salmonella typhi*, and *Shigella dysenteriae* were not susceptible to 2-Medpy-3-CN even at 64 µg·mL^−1^ (highest concentration tested). In addition to its broad-spectrum antibacterial activity, the compound also exhibited excellent antifungal activity against all five *Candida* strains tested. The MICs for *Candida* spp. ranged from 0.25 to 2 µg·mL^−1^. *Candida glabrata* was susceptible to 2-Medpy-3-CN at 0.25 µg·mL^−1^ and *Candida tropicalis* at 2 µg·mL^−1^. The highest bactericidal and fungicidal concentrations that inhibited the growth of bacteria (MBC) and fungi (MFC) were 64 µg·mL^−1^ and 4 µg·mL^−1^, respectively.

### 2.4. Time-to-Kill Assay

**Figure 2:**
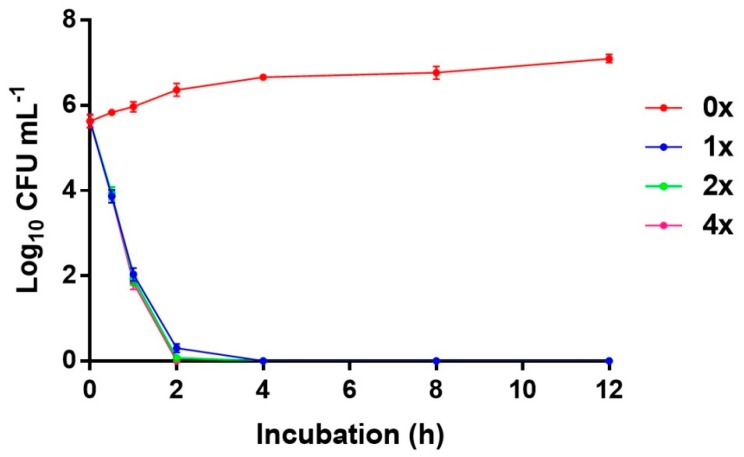
Effect of 2-Medpy-3-CN on the viability of methicillin-resistant *Staphylococcus aureus* (MRSA) grown in BHI at concentrations of 1×, 2×, and 4× MIC with control (0× MIC). MIC—minimum inhibitory concentration; CFU—colony-forming units.

Studies on the bacterial killing kinetics of 2-Medpy-3-CN on MRSA showed that the growth control in brain heart infusion(BHI) without any antibiotics (non-treated) maintained viability for 24 h, while MRSA treated with the test antibacterial compound at 1×, 2×, and 4× MIC showed significant reduction in growth, indicating that 2-Medpy-3-CN is strongly bactericidal in killing >90% of the cells at 2 h post-treatment. The compound did not exhibit a concentration-dependent activity, and no significant difference in log_10_ colony-forming units (CFU)·mL^−1^ reduction was observed. It was observed that, with an initial inoculum of ~10^6^ CFU·mL^−1^, 2-Medpy-3-CN (1×, 2×, and 4× MIC) declined the growth of MRSA as the incubation time increased from 0 to 2 h. The average reductions in CFUs were found to be 1.8-log_10_, 2.1-log_10_, and 1.8-log_10_ decreases in CFU·mL^−1^ at 0.5, 1, and 2 h post-treatment, respectively (Figure 2).

## 3. Discussion

The 2-Medpy-3-CN compound was isolated as a yellow-colored oil (later formed as yellow crystals). The LC–MS data displayed a molecular mass of 183.00471 *m/z*, signifying a molecular formula of C_7_H_6_N_2_S_2_, and the spectrum also gave the isotype motif for two sulfur atoms. The ^1^H-NMR spectrum (Table 5; Figure S1, Supplementary Materials) showed a deshielded methyl singlet at δ 2.60 and the presence of four hydrogens represented a characteristic ABCD aromatic structure/scheme. The ^13^C-NMR and DEPT-135 spectra (Table 5; Figures S2 and S8, Supplementary Materials) showed the presence of three methine aromatic carbons at δ 152.18–120.58, a deshielded quaternary carbon at δ 120.58S, and a methyl carbon at δ 23.23. This deshielded nature of the hydrogen atom (δ 8.77, H-6) of 2-Medpy-3-CN points out the phenomenon of a typical coupling of an alpha hydrogen to the nitrogen of a pyridine structure [27]. Hence, this pattern of coupling and correlation strongly underscores the possibility that 2-Medpy-3-CN could be a pyridine ring-containing natural compound.

Correlation spectroscopy (COSY) revealed the correlations of the aromatic hydrogens of 2-Medpy-3-CN (Figure S6, Supplementary Materials). In particular, the double-doublet of H-3 (δ 8.77) was coupled to a H-4 triplet (δ 7.89), which finally coupled to αH-6 doublet (δ 7.26). At δ 2.60, the presence of a methyl group appeared as a singlet featuring without couplings, indicating that it belongs to the aromatic nucleus. Its selected appearance in the ^1^H spectrum at 2.60 ppm determined that it was deshielded slightly and was attached to one of the sulfur atoms (apparently as a heteroatom). Heteronuclear multiple bond correlation spectroscopy (HMBC) showed the weak correlation (^4^*J*) between the methyl hydrogens and the C-2 quaternary carbon (HSQC), which suggested its terminal position on a disulfide side-chain α to the nitrogen (Figures S3 and S4, Supplementary Materials). Stereochemistry on the nuclear Overhauser spectroscopy (NOESY) effect of methyl hydrogens to H-3 was also determined (Figure S5, Supplementary Materials).

The IR data indicated the presence of nitrile (2221 cm^−1^) and pyridine (1544, 1430, 1230, 731 cm^−1^) moieties (Figure S10, Supplementary Materials). The molecular formula of 2-Medpy-3-CN was C_7_H_6_N_2_S_2_ (Figure S11, Supplementary Materials), which left the placement of the nitrile group in the skeleton of 2-Medpy-3-CN. Thus, the structure of the pure fraction D_3/6e_ was determined as 2-(methyldithio)pyridine-3-carbonitrile (Figure 1). Previously, pyridine-*N*-oxide compounds were reported from *A. stipitatum* [25,26,28]; however, this is the first report on a naturally existing pyridine compound with a nitrile as a functional group from *A. stipitatum*.

The promising antibacterial activities of pyridine and thiopyridines derivatives ere adequately reported [29,30,31]. The 2-Medpy-3-CN compound demonstrated outstanding potency with low MIC values ranging from 0.5 to >64 µg·mL^−1^. The MICs of 2-Medpy-3-CN are consistent with the earlier data on pyridine based compounds from other research groups [25,26,28]. O’Donnell and his colleagues were the first to isolate and report pyridine-*N*-oxide alkaloids with disulfide functional groups from natural sources [26]. Three compounds named 2-(methyldithio)pyridine-*N*-oxide, 2[(methylthiomethyl)dithio]pyridine-*N*-oxide, and 2,2’-dithio-bis-pyridine-*N*-oxide (dipyrithione) were reported. Of the three compounds, 2-(methyldithio)pyridine-*N*-oxide and 2[(methylthiomethyl)dithio]pyridine-*N*-oxide were shown to possess strong antibacterial activity against fast-growing *Mycobacterium* sp., MRSA, and MDR variants of *S. aureus* with MICs of 0.5–8 µg·mL^−1^. These results were in accordance with the present study results where an MIC of 4 µg·mL^−1^ was observed for MSSA and MRSA strains. Following O’Donnell’s discovery of pyridine *N*-oxide compounds, Krejčová and his colleagues reported the anti-inflammatory and neuroprotective effects of five naturally occurring sulfur-containing pyridine-*N*-oxides from *A. stipitatum* [28]. The compound of the present study, 2-Medpy-3-CN, harbors nitrile as a functional group, which is another likely perception for its strong antimicrobial properties. Carbonitrile-containing semisynthetic derivatives of pyridine molecules exhibit strong antibacterial activity [32,33,34]. In addition to antibacterial activity, 2-Medpy-3-CN also exhibited very strong anticandidal activity, attributed to a broad-spectrum antimicrobial activity at a very low MIC level of 2 µg·mL^−1^ for *C. tropicalis*. Semisynthetic pyrimidine derivatives with carbonitrile as the functional group at the fifth position were reported to have strong antibacterial and antifungal activities at 12.5 µg·mL^−1^ [33,35]. The extreme potency of 2-Medpy-3-CN foreshadows its possibility in clinical use.

Time kill study was used to explore the bactericidal activity of 2-Medpy-3-CN using MRSA American Type Culture Collection (ATCC) 43300. The killing kinetics of 2-Medpy-3-CN demonstrated time-dependent killing instead of a dose-dependent pattern. At 1× MIC (4 µg·mL^−1^), 2-Medpy-3-CN effected a time-dependent reduction in the viability of the tested bacterial strain. Increasing the concentration of 2-Medpy-3-CN did not show any change in the bactericidal action. The killing rates were very much similar at 2× and 4× MIC, with 1.8-log_10_, 2.1-log_10_, and 1.8-log_10_ reductions in the number of CFU·mL^−1^ after 0.5, 1, and 2 h of incubation, respectively. For the first time, our study reports the killing kinetics or the rate of killing of pyridine compounds on MRSA. A recent study on novel 2-thiopyridines against actively growing and dormant cells of *M. tuberculosis* for seven days at 10 µg·mL^−1^ showed more than 2-log-unit killing effect [31]. Remarkably, the tested concentration and the CFU reductions were similar to the results of the present study. Testing 2-Medpy-3-CN on a diverse panel of antibiotic-resistant *S. aureus* isolates at various MICs disclosed the consistency of anti-MRSA activity.

Absorption, distribution, metabolism, and excretion (ADME) properties were used to determine the “drug-like” characteristics of the ligand molecule. ADME predictions can be used to focus lead optimization efforts in enhancing the desired properties of a given compound. The expected ADME property of the tested compound was evaluated with the QikProp module of Schrodinger. Almost all the predicted properties of the tested compound were in the range as predicted by QikProp for 100% (>80% is high) of known oral drugs, and the compound also satisfies Lipinski’s rule of five to be considered as having drug-like potential.

## 4. Materials and Methods

### 4.1. Chemistry

#### 4.1.1. Plant Material and Preparation of Extracts

The source and the preparation of the extract were described earlier [22]. Briefly, dried bulbs (5 kg) of *A. stipitatum* were ground into fine powder and extracted with 10 L of dichloromethane for 72 h (for each solvent) by maceration at room temperature. The extract was filtered through Whatman No.1 paper to remove solid plant materials, and the filtrate was dried under vacuum (BÜCHI Rotovapor R-200, Flawil, Switzerland) at 40 °C. Upon filtration and solvent volatilization, the dichloromethane extract yielded 164 g (3.28%) of residue.

#### 4.1.2. General Experimental Procedures

Analytical-grade solvents, including hexane and dichloromethane used for extraction and isolation, were purchased from (Merck KGaA, Darmstadt, Germany). Column chromatography was carried out using silica gel Merck 7734 (70–230 mesh ASTM) and Merck 9385 (230–400 mesh ASTM). Thin-layer chromatography (TLC) analyses were carried out on Merck silica gel DC-plastikfolien 60 F254 plastic sheets and TLC spots were visualized using a UV lamp at 254 and 366 nm, respectively. Melting point was recorded using an electrothermal digital melting point apparatus. Ultraviolet (UV) and IR spectra were recorded on a Varian UV–visible light (UV–Vis) 50 and Perkin-Elmer 100 Fourier-transform infrared (FTIR) spectrophotometers, respectively. The isolated compound was dissolved in CDCl_3_ (deuterated chloroform), and the NMR spectra (1D and 2D NMR) were recorded on a Bruker AVANCE 500 Ultrashield NMR spectrometer. The molecular weight of the isolated compound was determined on an Agilent 1290 Infinity II LC system (Agilent Technologies, Santa Carla, CA, USA) coupled to a 6520 Q-TOF tandem mass spectrometer for separation. The chemical shifts (δ) were determined from the residual solvent peaks.

#### 4.1.3. Isolation of Potential Antimicrobial Compounds

**Scheme 1:**
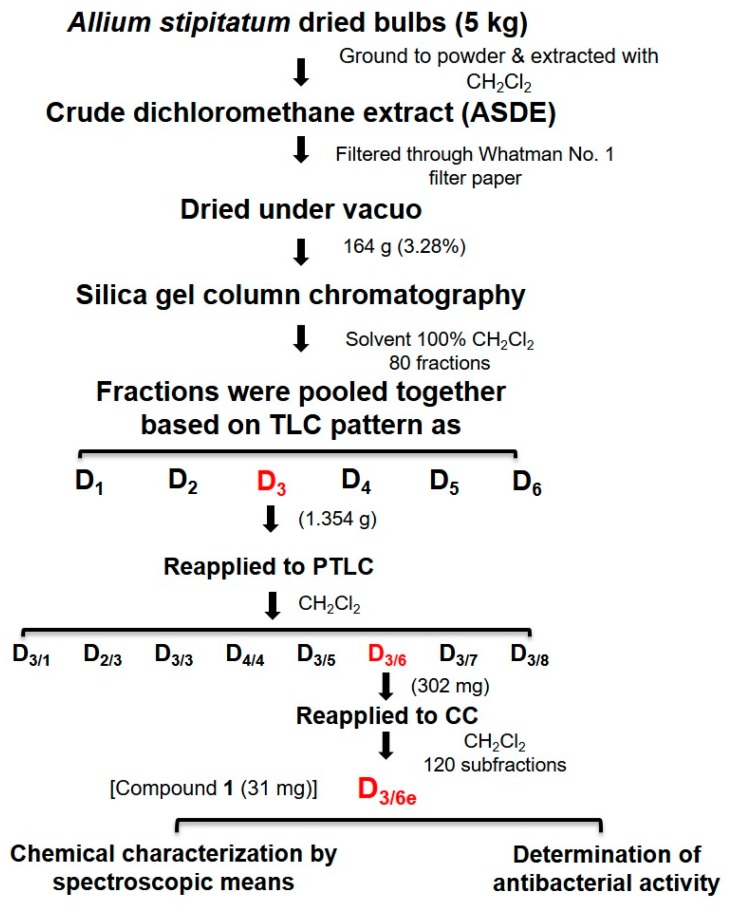
Purification scheme of fraction D_3/6e_ from the bulbs of *Allium stipitatum*.

TLC analysis of the *Allium stipitatum* dichloromethane (ASDE) extract showed the presence of large, clear spots when visualized under a UV chamber and iodine staining. The crude dichloromethane extract of *A. stipitatum* bulbs (164 g) was subjected to silica gel column chromatography (CC) using CH_2_Cl_2_ as the mobile phase. A total of 80 fractions (50 mL in volume) were collected in 100-mL conical flasks. All fractions were subjected to TLC analysis. Fractions that showed similar TLC patterns were pooled together which yielded six major fractions (D_1_–D_6_). The six fractions (D_1_–D_6_) were tested for their antibacterial activity against MRSA. Fraction D_3_ (1.354 g) which exerted remarkable antibacterial activity, was subjected to preparative thin-layer chromatography (PTLC) using CH_2_Cl_2_ as the mobile phase to yield eight subfractions (D_3/1_–D_3/8_). Subfraction D_3/6_ (302 mg) was further subjected to column chromatography (CC) using CH_2_Cl_2_ as the mobile phase to yield 120 fractions (10 mL in volume). Fractions were pooled together based on TLC patterns. Subfraction D_3/6e_ (31 mg) was identified as a pure fraction and subjected to NMR and mass analysis. The purification scheme of fraction D_3/6e_ is illustrated in Scheme 1. At each purification step, the fractions and subfractions were tested for their antibacterial activity using the micro-broth dilution method.

### 4.2. Biological Evaluation with Methicillin-Resistant S. aureus (MRSA)

#### 4.2.1. Test Microorganisms

MRSA ATCC 43300 was used as model organism to screen for the antimicrobial activity. For the anti-MRSA activity, the confirmed compound was further screened for broad-spectrum activity against other pathogenic bacteria including *Acinetobacter baumannii*, *A. iwoffii*, *Enterobacter* sp., *Escherichia coli*, *Staphylococcus aureus*, *Pseudomonas aeruginosa*, *Salmonella typhi*, *Shigella dysenteriae,* and *Stenotrophomonas maltophilia* through MIC (minimum inhibitory concentration) and MBC (minimum bactericidal concentration) determinations. The compound was also tested for antifungal activity against five different pathogenic forms of *Candida* species, namely *C. albicans*, *C. glabrata*, *C. krusei*, *C. parapsilosis*, and *C. tropicalis*, for MIC and MFC (minimum fungicidal concentration).

#### 4.2.2. MIC, MBC, and MFC Measurements

For the determination of MIC, MBC, and MFC, the compound was dissolved in 10% dimethyl sulfoxide (DMSO) with a graded concentration in the range of 64–0.125 µg·mL^−1^ with MHB for bacteria and Roswell Memorial Park Institute (RPMI-1640) medium for *Candida* strains. The MIC of the compound was determined using microbroth dilution method as described for bacteria and yeast [36,37]. Fluconazole was used as an antifungal standard and its MIC was determined using the Etest method. Appropriate antibiotic controls were included as positive controls based on the recommendations by the Clinical Laboratory Standards Insititute CLSI (2012). DMSO (10%) was used as a negative control and broth cultures without antibiotics served as growth controls.

#### 4.2.3. Time-To-Kill Assay for Detecting the Bactericidal Effect of the Compound for MRSA

The killing kinetics of the compound on MRSA ATCC 43300 at 0×, 1× (4 µg·mL^−1^), 2× (8 µg·mL^−1^), and 4× (16 µg·mL^−1^) MIC was determined according to the method described previously [38] with some modifications. Bacterial and yeast suspensions were diluted to 1 × 10^6^ CFU·mL^−1^. The compound concentrations were adjusted to 1× MIC, 2× MIC, and 4× MIC. Cultures treated with the varying concentrations of the compound were incubated at 37 °C for 0, 0.5, 1, 2, 4, 8, and 12 h. Aliquots of 100 µL were pipetted out from each tube at each time point, serially diluted in phosphate-buffered saline (PBS) and spread onto Mueller Hinton Agar MHA plates. Tubes without the compound served as growth controls (0×). The plates were incubated at 37 °C for 24 h followed by the enumeration of the colonies. Killing curves were constructed by plotting the log_10_ CFU·mL^−1^ versus time over a 24 h time period. A decline in bacterial/fungal growth to ≥3log_10_ CFU·mL^−1^ from the initial inoculum was considered to be bactericidal/fungicidal.

### 4.3. Prediction of ADME Properties

Schrodinger’s QikProp module is an accurate, quick, and easy-to-use absorption, distribution, metabolism, and excretion (ADME) prediction program that was designed to produce certain ADME-related descriptors. QikProp consists of two modes: normal mode and fast mode. In the present study, the QikProp program, version 3.4 was run in normal processing mode with default options [39]. To set up the calculation, a pose viewer file (generated after docking with Glide) was used to consider the receptor and source of ligands. The program was run (with default options) and reasonable descriptors were produced.

### 4.4. Statistical Analysis

All experiments were performed in triplicate except for the time-to-kill assay. Differences between the treated and untreated (control) groups were analyzed using GraphPad Prism 5.0. One-way ANOVA was performed and a Dunnett’s post hoc test was used for the comparison of multiple means. Significance was set at a *p*-value of <0.05.

## 5. Conclusions

The present study isolated a novel broad-spectrum antimicrobial compound 2-(methyldithio)pyridine-3-carbonitrile. The 2-Medpy-3-CN compound showed excellent antimicrobial activity against a panel of bacterial and fungal pathogens. The compound inhibited the growth of Gram-positive and Gram-negative pathogens along with *Candida* species at MICs ranging from 0.5 to >64 µg·mL^−1^. The 2-Medpy-3-CN compound was strongly bactericidal against MRSA in less than 2 h post-treatment. Further studies are vital to identify the drug target and mechanism of action for this highly potent antimicrobial compound.

## Figures and Tables

**Table 1:** *Allium stipitatum* dichloromethane (ASDE) fractions and their respective minimum inhibitory concentrations (MICs) against methicillin-resistant *Staphylococcus aureus* (MRSA) American Type Culture Collection (ATCC) 43300.

Fraction	Weight (g)	Physical Appearance	MIC for MRSA (µg·mL^−1^)
D_1_	0.636	Yellow liquid	No activity
D_2_	1.055	Brown solid	128
D_3_	1.354	Brown gum	32
D_4_	1.875	Brown gum	256
D_5_	1.206	Dark-yellow solid	No activity
D_6_	0.749	Yellow solid	No activity

**Table 2:** ASDE subfractions of D_3_ and their respective MICs against MRSA ATCC 43300.

Fraction	Weight (g)	Physical Appearance	MIC for MRSA (µg·mL^−1^)
D_3/1_	0.153	Yellow gum	No activity
D_3/2_	0.114	Brown solid	No activity
D_3/3_	0.196	Dark brown solid	No activity
D_3/4_	0.115	Brown gum	32
D_3/5_	0.087	Brown gum	16
D_3/6_	0.302	Brown gum	16
D_3/7_	0.180	White solid	32
D_3/8_	0.242	White solid	No activity

**Table 3:** ASDE subfractions of D_3/6_ and their respective MICs against MRSA ATCC 43300.

Fraction	Weight (mg)	Physical Appearance	MIC for MRSA (µg·mL^−1^)
D_3/6a_	17	Brown gum and white solid	No activity
D_3/6b_	35	Brown gum and white solid	No activity
D_3/6c_	48	Brown gum and yellow solid	No activity
D_3/6d_	65	Yellow liquid	32
D_3/6e_	31	Yellow liquid	4
D_3/6f_	28	Pale-yellow solid	No activity

**Table 4:** One-dimensional (1D) and two-dimensional (2D) NMR spectral data of 2-(methyldithio)pyridine-3-carbonitrile (2-Medpy-3-CN).

Position	δ*_H_* (*J* in Hz)	δ_C_, Type	DEPT 90	DEPT 135	COSY	NOESY	HMBC	HSQC
2		162.18, C					4,6	
3		107.87, C					4,5,6	
4	7.89, 1H, dd (1.5, 7.5 Hz)	141.02, CH	141.04, CH	141.04, CH	5,6	5	5,6	4
5	7.26, 1H, dd (4.5, 7.5 Hz)	120.58, CH	120.58, CH	120.58, CH	4,6	4,6	4,6	5
6	8.77, 1H, dd (1.5, 4.5 Hz)	152.87, CH	152.88, CH	152.89, CH	4,5	5	4,5	6
–CN		114.96, C					4,5,6	
–CH_3_	2.60, 3H, s	23.23, CH_3_		23.23		7	6	–CH_3_

**Table 5:** Minimum inhibitory concentration (MIC), minimum bactericidal concentration (MBC), and minimum fungicidal concentration (MFC) determinations of 2-Medpy-3-CN against human pathogenic bacteria and *Candida* spp. DMSO—dimethyl sulfoxide.

Microorganisms	Antimicrobial Activity			
	2-Medpy-3-CN		Antibiotic (µg·mL^−1^)	DMSO (10%) (20 µL Per Disk)
	MIC (µg·mL^−1^) ^a^	MBC (µg·mL^−1^) ^b^		
**Bacteria**				
*Acinetobacter baumannii*	4	8	≤4 (IMP)	-
*Acinetobacter iwoffii*	0.5	1	≤4 (IMP)	-
*Escherichia coli*	32	64	0.5–2 (TET)	-
Methicillin-resistant *Staphylococcus aureus*	4	8	0.5–2 (VAN)	-
Methicillin-sensitive *S. aureus*	4	16	≤8 (VAN)	-
*Pseudomonas aeruginosa*	>64	>64	>64 (TMP)	-
*Salmonella typhi*	>64	>64	<1 (CIP)	-
*Shigella dysenteriae*	>64	>64	≤8/4 (AM/CL)	-
*Stenotrophomonas maltophilia*	32	>64	≥2–38 (TP/SX)	-
**Yeast**				
*Candida albicans*	0.5	1	0.12 (FLU) ^c^	-
*Candida glabrata*	0.25	1	16 (FLU) ^c^	-
*Candida krusei*	1	4	1 (FLU) ^c^	-
*Candida parapsilosis*	0.5	2	0.5 (FLU) ^c^	-
*Candida tropicalis*	2	4	32 (FLU) ^c^	-

^a^ Determined using microbroth dilution method. ^b^ Determined using spread plate technique. ^c^ Determined using Etest method.

## Supplementary Materials

The following are available online at https://www.mdpi.com/1420-3049/24/6/1003/s1, ^1^H and ^13^C-NMR data and other related spectra for 2-Medpy-3-CN within this article, along with ADME toxicity data, can be found in the Supplementary Materials.
