# Supplementary material for: Bioactive 2-(Methyldithio)Pyridine-3-Carbonitrile from Persian Shallot (Allium stipitatum Regel.) Exerts Broad-Spectrum Antimicrobial Activity
Source: Molecules. 2019 Mar 13;24(6):1003. doi: 10.3390/molecules24061003 (PMC6471189; doi:10.3390/molecules24061003)
Supplement: Supplementary file 1 [file molecules-24-01003-s001.pdf]

Table S1 Nomenclature of *fab* genes and their biological functions.

Gene	Encoding enzyme	Short name	Function
fabA	*β*-hydroxyacyl-acyl carrier protein dehydratase	*β*-hydroxy ACP dehydratase (Bifunctional enzyme)	Catalyzes the dehydration of hydroxyl-ACP to *trans*-2-enoyl-ACP<br>Isomerizes *trans*-2-decenoyl-ACP to *cis*-3-decenoyl-ACP
fabB	*β*-ketoacyl-acyl carrier protein synthase I	*β*-ketoacyl-ACP synthase I (KAS I) (Condensation)	Condensation of malonyl-ACP with acyl-ACP<br>Elongates *cis*-unsaturated fatty acid
fabD	Malonyl-CoA-acyl carrier protein transacylase	Malonyl-CoA ACP transacylase (MCAT)	Transfers the malonyl group from malonyl-CoA to holo-acyl carrier protein (ACP)
fabF	*β*-ketoacyl-acyl carrier protein synthase II	*β*-Ketoacyl-ACP synthase II (KAS II)	Condensation of malonyl-ACP with acyl-ACP (Elongation condensing enzyme)
fabG	*β*-ketoacyl-acyl carrier protein reductase	*β*-ketoacyl ACP reductase (NADPH dependent reduction)	*β*-ketoester is reduced to a *β*-hydroxyacyl-ACP reductase
fabH	*β*-ketoacyl-acyl carrier protein synthase III	*β*-ketoacyl-ACP synthase III (KAS III)	Condenses malonyl-ACP with acetyl-CoA to form acetoacetyl ACP (Elongation)
fabI	Enoyl-acyl carrier protein reductase I	Enoyl ACP reductase (NADPH dependent reduction)	Conversion of *trans*-2-enoyl-ACP to acyl-ACP (final rate limiting step)
fabK	Enoyl-acyl carrier protein reductase II	Enoyl-ACP reductase II (Final rate-limiting step in FAS II)	Conversion of *trans*-2-enoyl-ACP to acyl-ACP (final rate limiting step)
fabL	Enoyl-acyl carrier protein reductase III	Enoyl-ACP reductase II (Final rate-limiting step in FAS II)	Conversion of *trans*-2-enoyl-ACP to acyl-ACP (final rate limiting step)
fabZ	*β*-hydroxyacyl-acyl carrier protein dehydratase	*β*-hydroxy ACP dehydratase	Formation of *cis*-3-decenoyl-ACP<br>Catalyzes the dehydration of hydroxyl-ACP to *trans*-2-enoyl-ACP

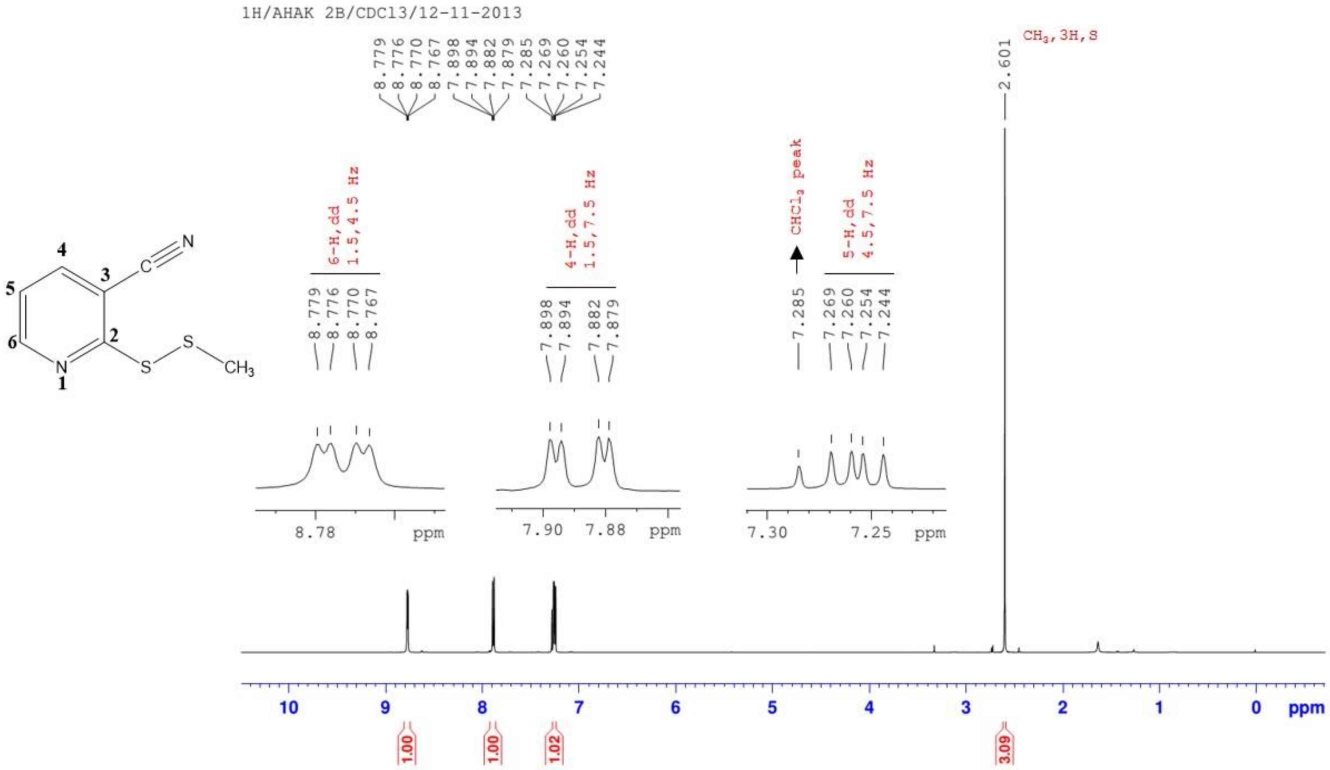

Figure S1 ^{1}H NMR Spectra of 2-Medpy-3-CN

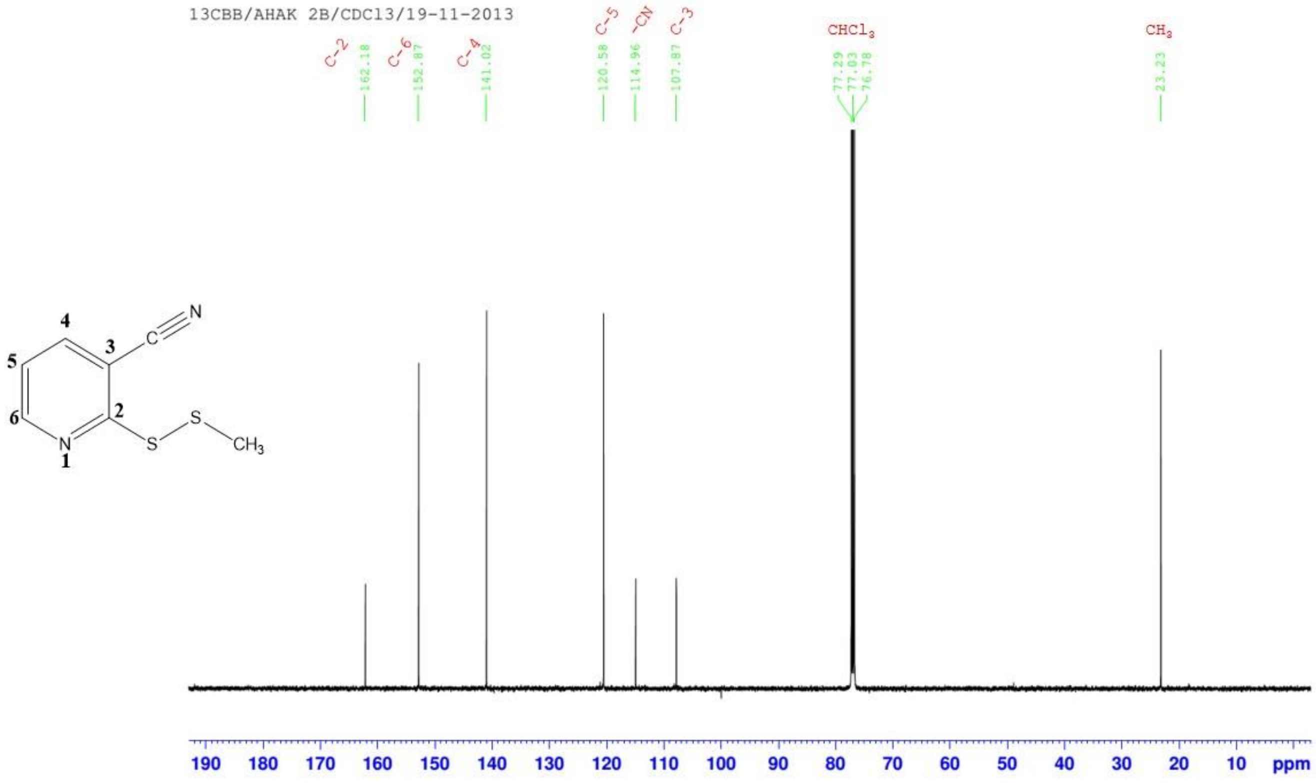

Figure S2 ^{13}C NMR Spectra of 2-Medpy-3-CN

Commented [P11]: C-2 EXPECTED TO BE AT 180

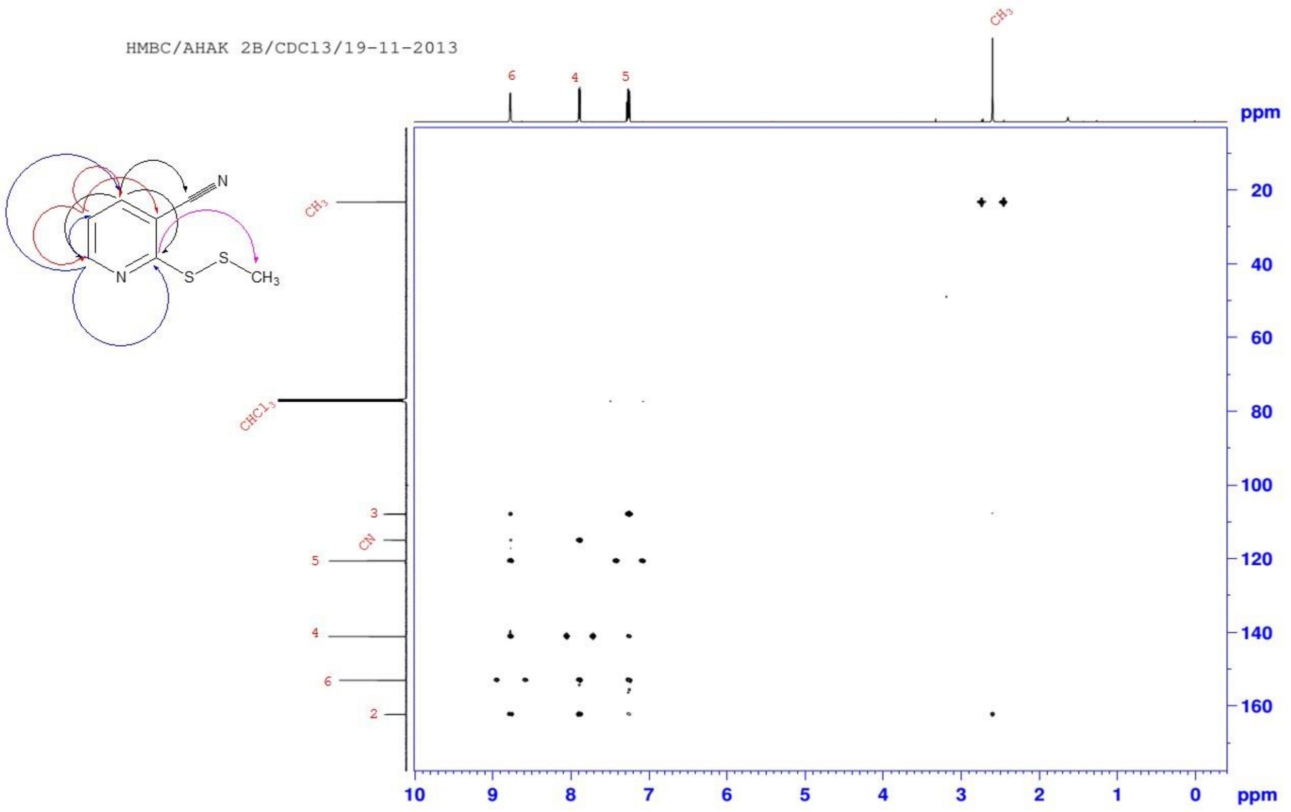

Figure S3a HMBC NMR Spectra of 2-Medpy-3-CN

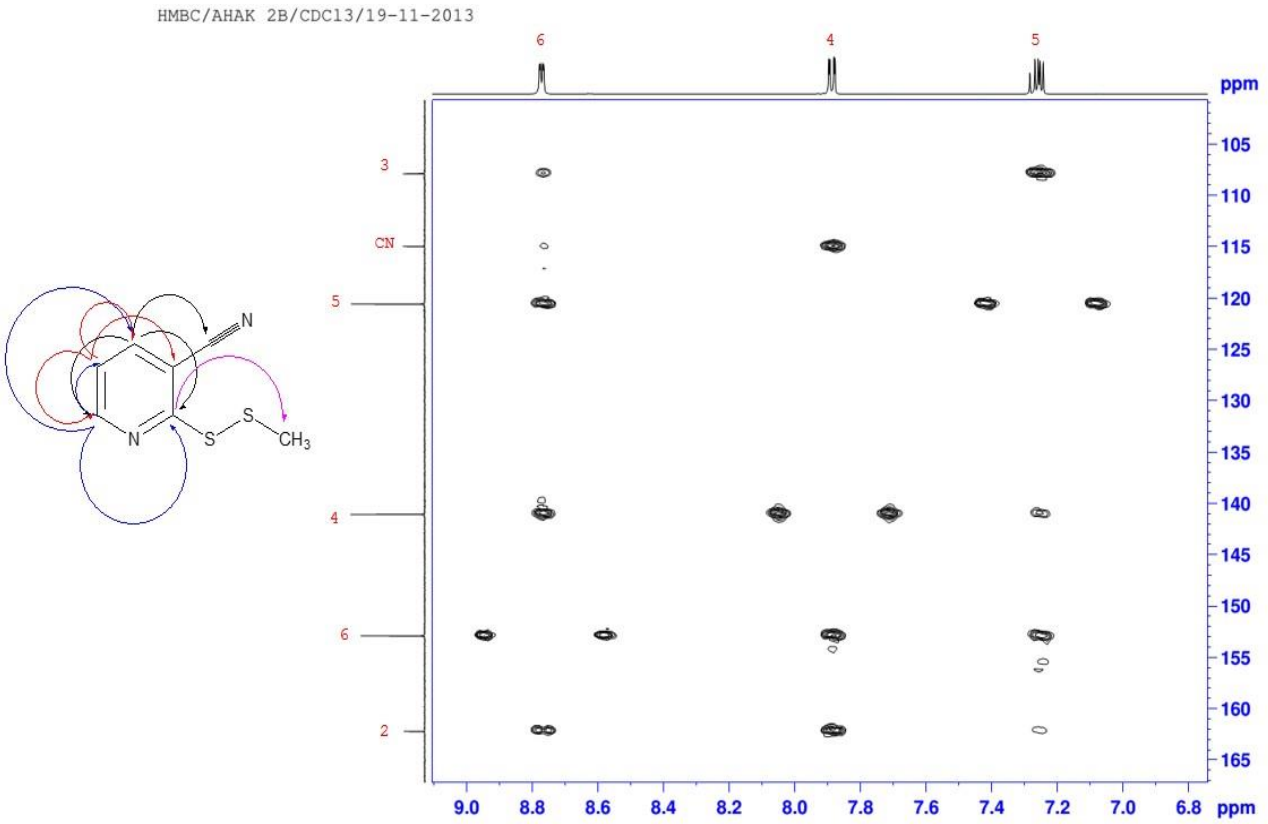

Figure S3b HMBC NMR Spectra of 2-Medpy-3-CN

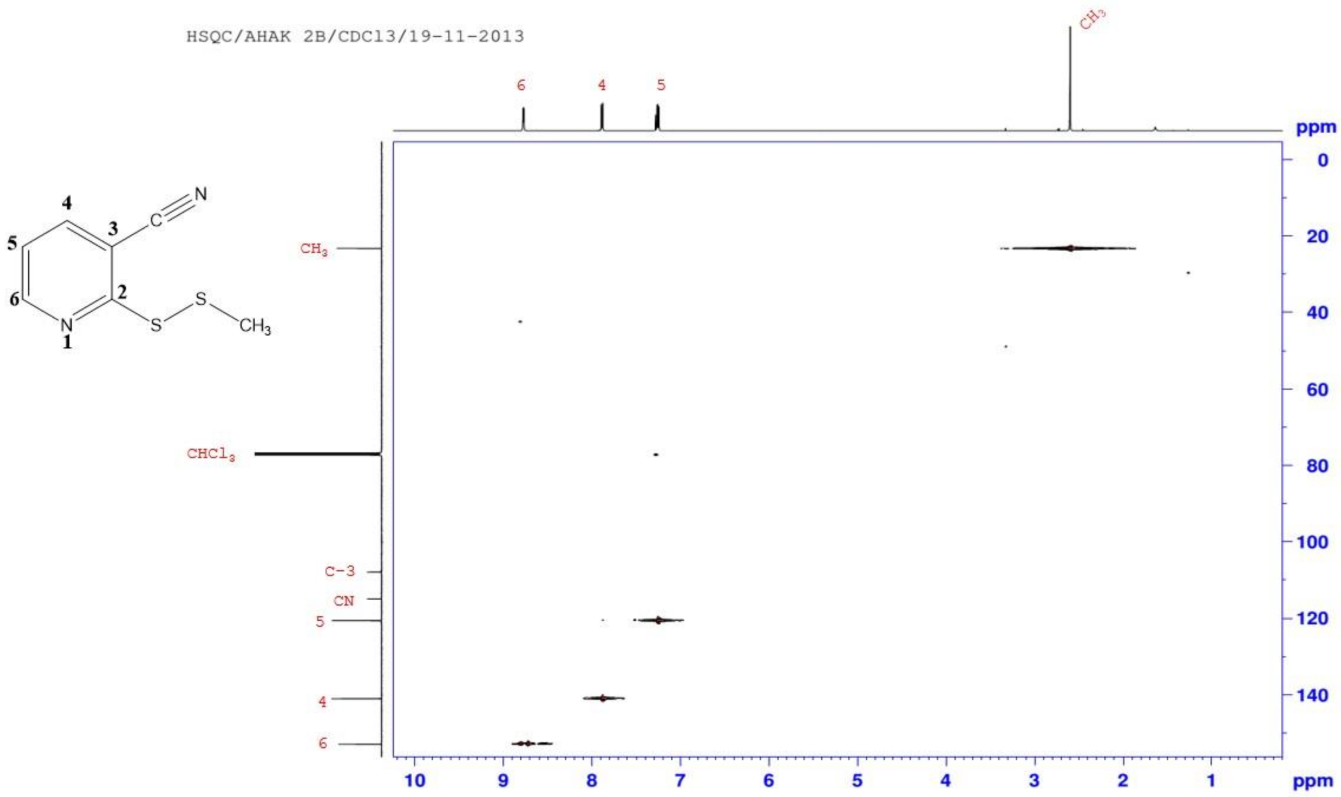

Figure S4 HSQC NMR Spectra of 2-Medpy-3-CN

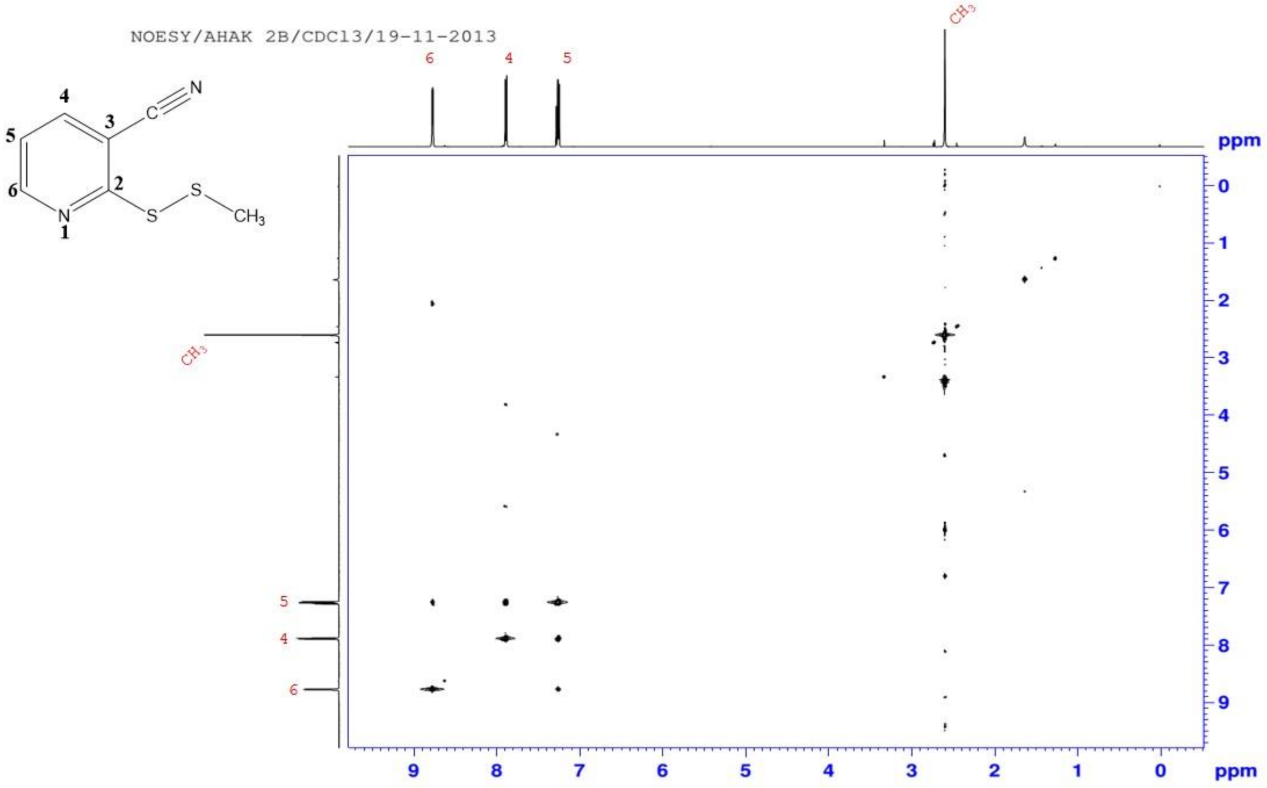

Figure S5 NOESY NMR Spectra of 2-Medpy-3-CN

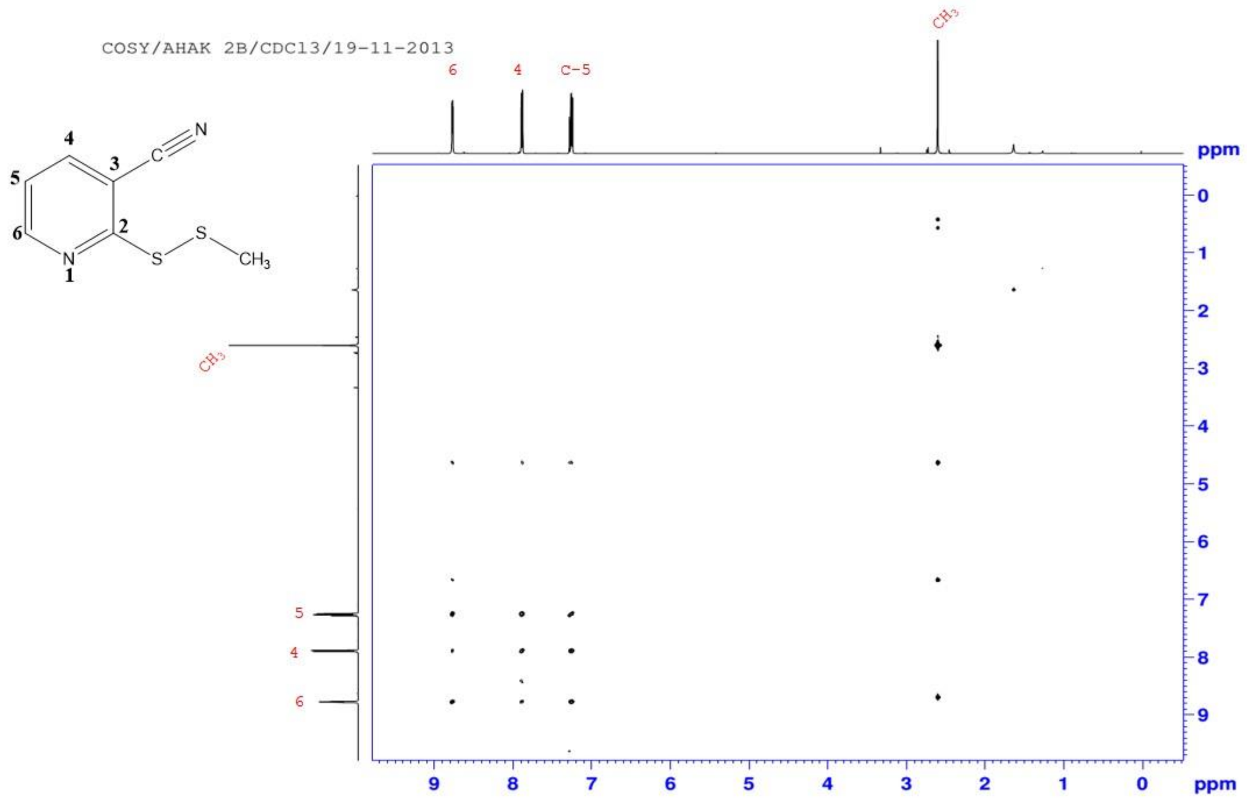

Figure S6 COSY NMR Spectra of 2-Medpy-3-CN

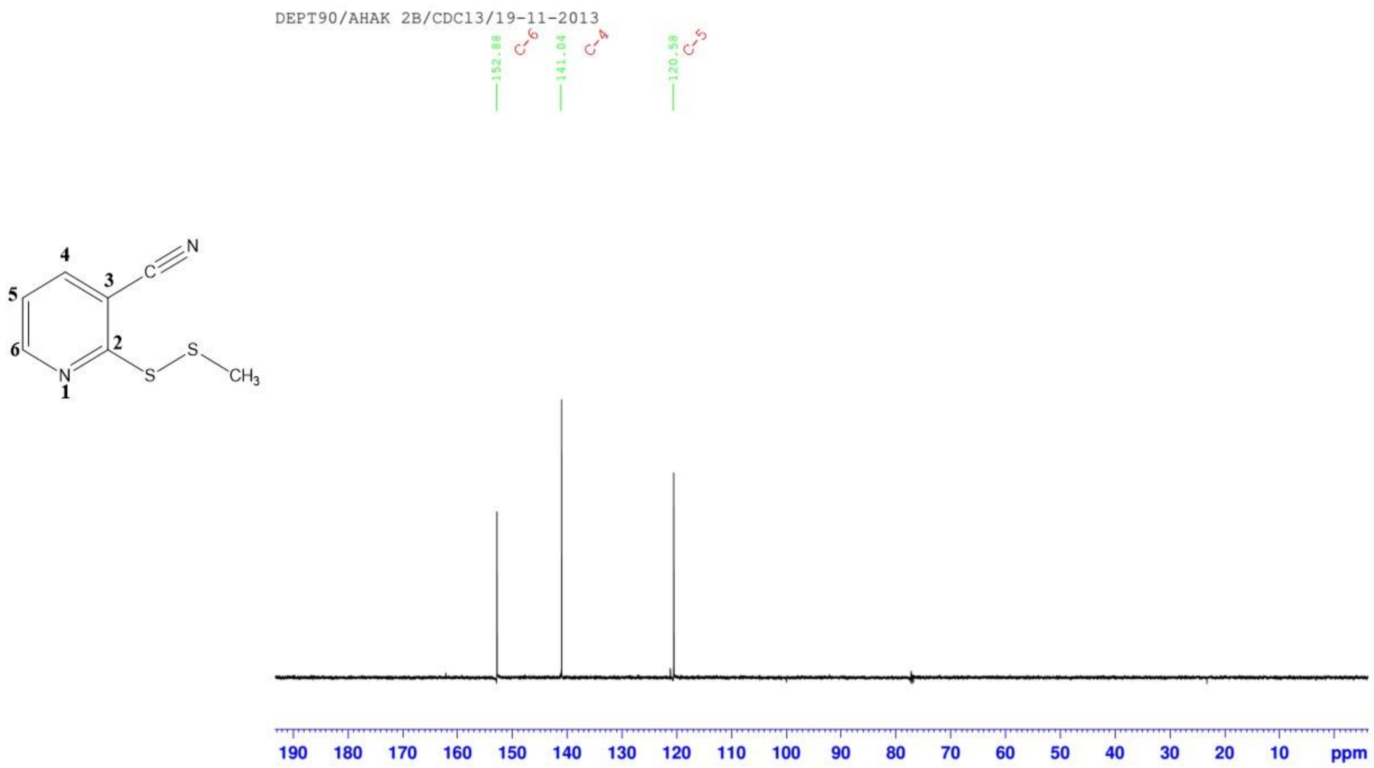

Figure S7 DEPT90 NMR Spectra of 2-Medpy-3-CN

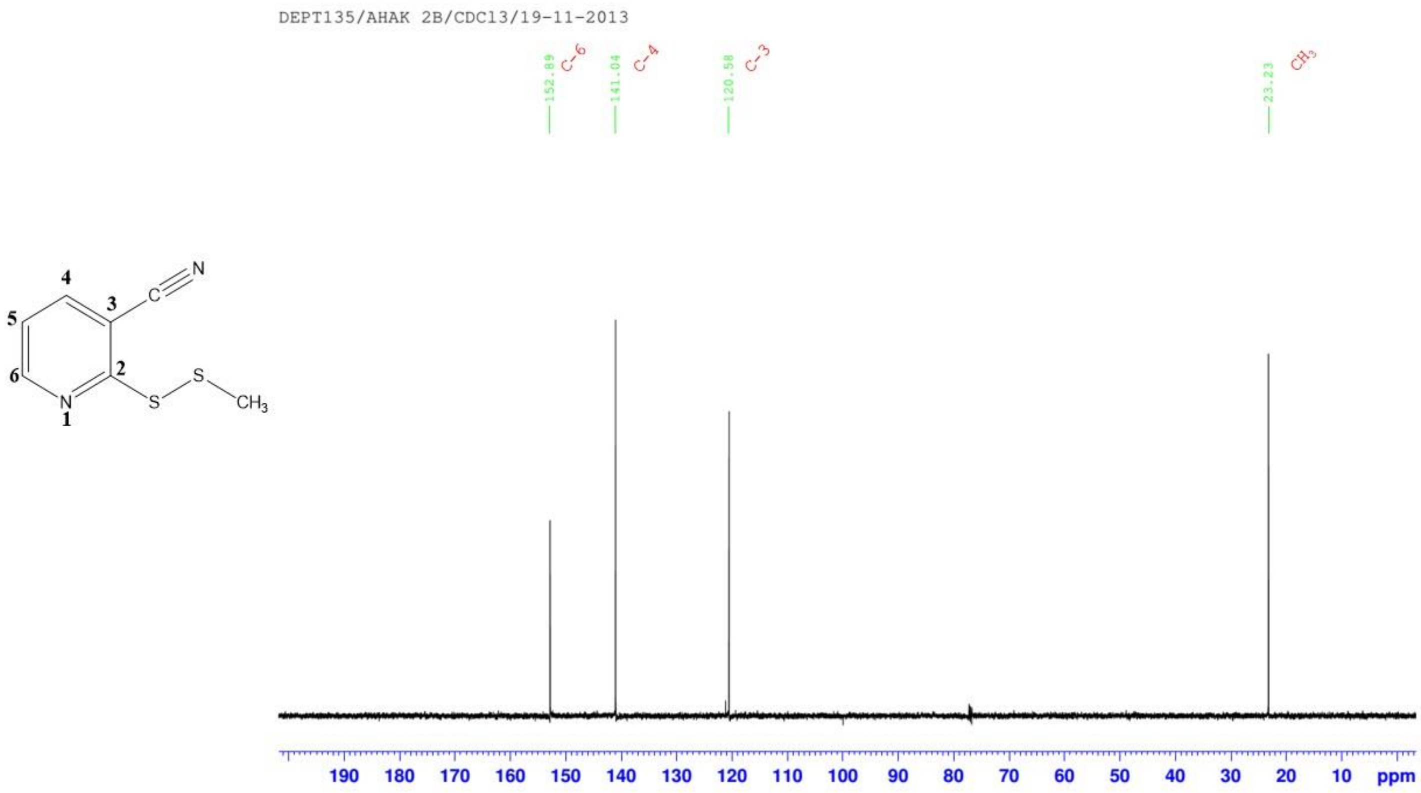

Figure S8 DEPT135 NMR Spectra of 2-Medpy-3-CN

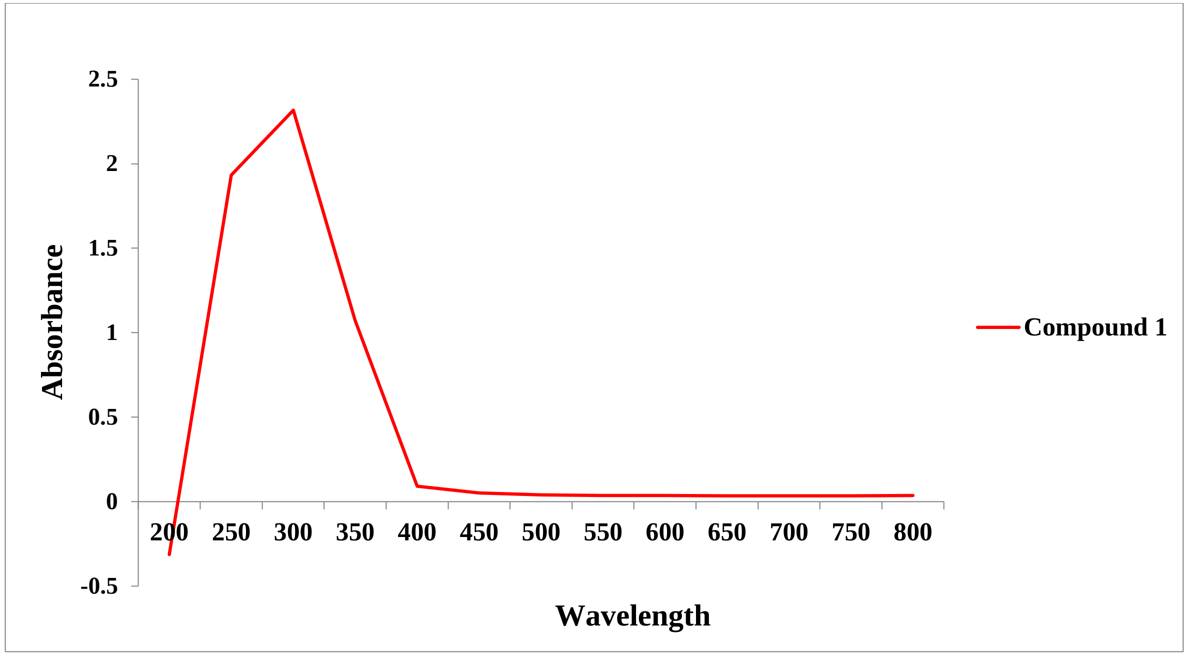

Figure S9 UV-Vis Spectra of 2-Medpy-3-CN

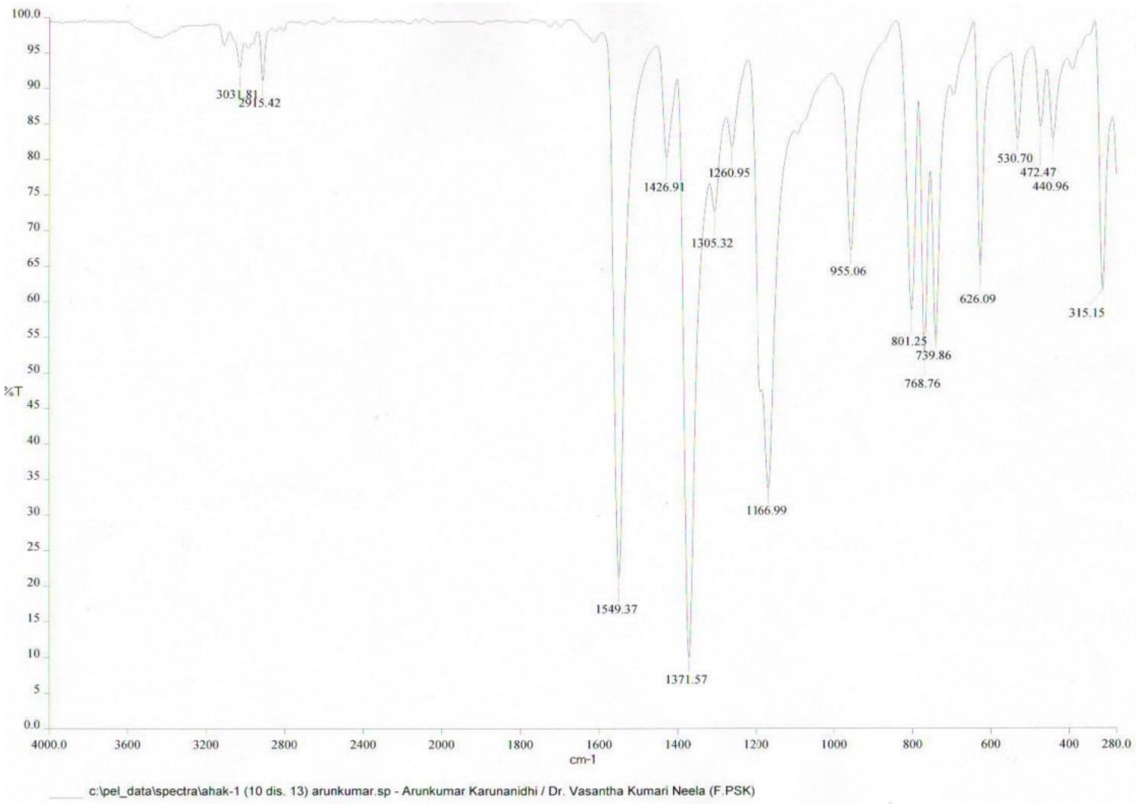

Figure S10 FT-IR Spectra of 2-Medpy-3-CN

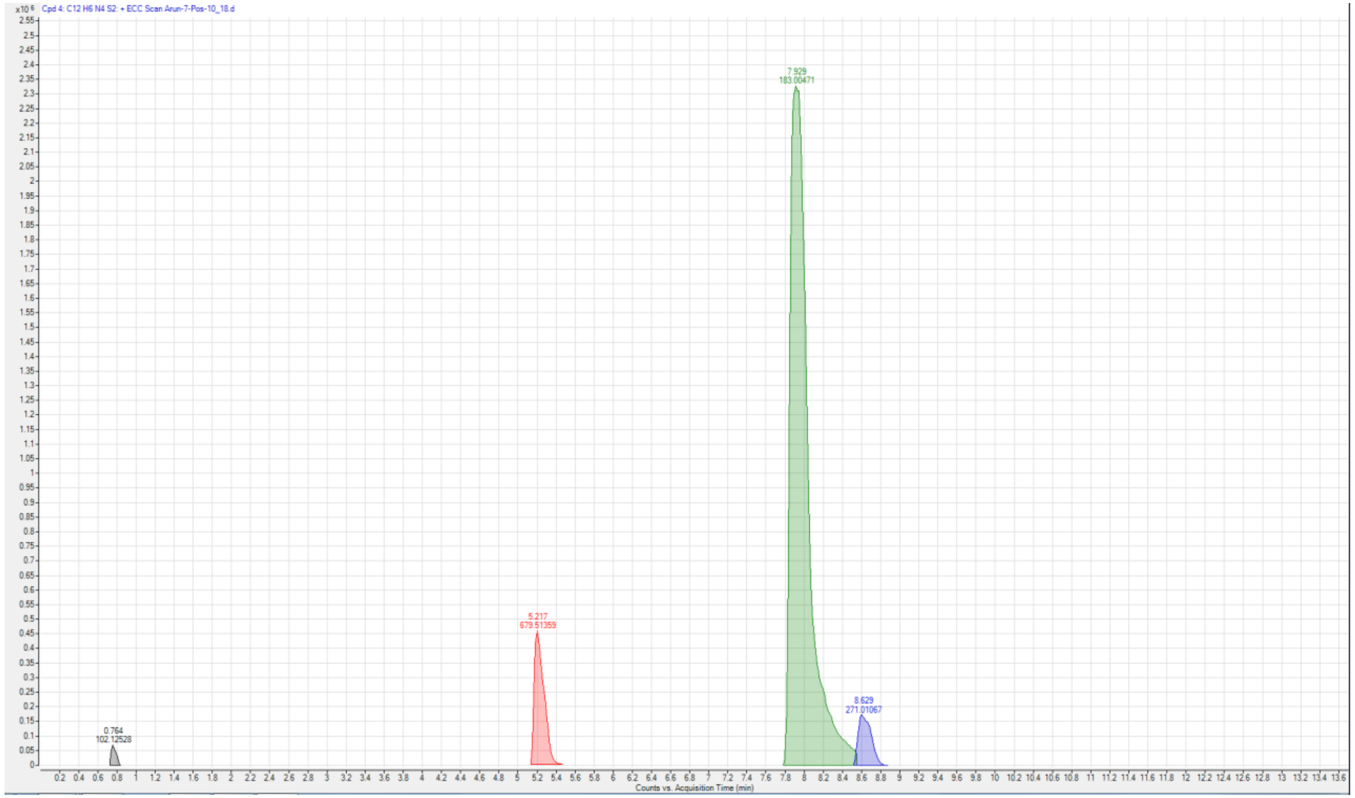

Figure S11 LC-MS Spectra of 2-Medpy-3-CN

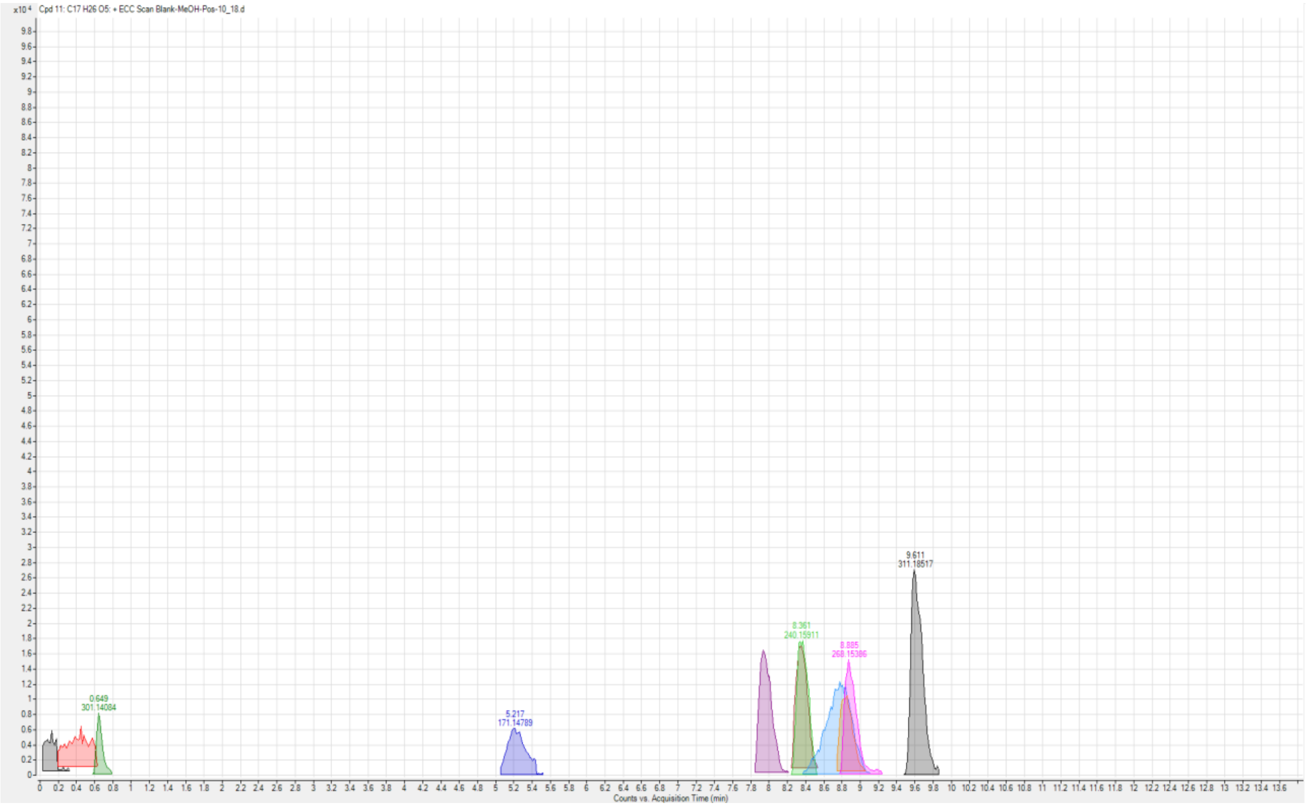

Figure S12 LC-MS Spectra of Blank (Control)

Table S2 ADME properties of 2-Medpy-3-CN

**Molecule**	**2-Medpy-3-CN**
#stars	1
#amine	0
#amidine	0
#acid	0
#amide	0
#rotor	3
#rtvFG	1
CNS	0
mol-MW	182.258
dipole	6.489
SASA	388.423
FOSA	83.331
FISA	71.636
PISA	155.084
WPSA	78.371
volume	609.021
donorHB	0
accptHB	3
dip^2/V	0.069148
ACxDN^.5/SA	0
glob	0.894573
QPpolrz	17.744
QPlogPC16	5.964
QPlogPoct	8.06
QPlogPw	4.517
QPlogPo/w	2.112
QPlogS	-2.618
CIQPlogS	-2.912
QPlogHERG	-3.978
QPPCaco	2072.879
QPlogBB	-0.022
QPPMDCK	2923.125
QPlogKp	-2.005
IP(eV)	9.446
EA(eV)	2.198
#metab	1
QPlogKhsa	-0.544
Human Oral Absorption	3
Percentage Human Oral Absorption	100
Safluorine	0
SAamideO	0
PSA	34.251
#NandO	2
Rule of Five	0
#ring atoms	6
# in 34	0
# in 56	6

# noncon	0
# nonHatm	11
Rule of Three	0
Jm	2.483
